# Genotype distribution and the relative risk factors for human papillomavirus in Urumqi, China

**DOI:** 10.3892/etm.2013.1073

**Published:** 2013-04-23

**Authors:** ZHIFANG CHEN, WEI MENG, RONG DU, YUEJIE ZHU, YI ZHANG, YAN DING

**Affiliations:** 1 Xiangya Hospital, Central South University, Changsha, Hunan 410000;; 2 Gynecology Department, First Affiliated Hospital of Xinjiang Medical University, Urumqi, Xinjiang 830054, P.R. China

**Keywords:** human papillomavirus, genotype distribution, cervical cancer

## Abstract

The aim of this study was to investigate human papillomavirus (HPV) infection and HPV genotype distributions in Urumqi, Xinjiang, China. The related risk factors for high-risk HPV infection was also analyzed. A stratified cluster sampling method was used for the population-based cervical cancer screening of women aged 18–69 years in the Urumqi Saybagh district. Exfoliated cervical cell samples were collected for liquid-based cytology detection and HPV genotyping DNA microarrays. Education level, number of sexual partners, condom use and occupation were used in the multivariate analysis model. The HPV infection rate of women working in service industries was significantly higher compared with those of white-collar workers, community residents and migrant workers. The 35–44-year-old migrant worker group had the highest HPV infection rates among all of the groups in the three different age ranges. The number of marriages, education level, smoking history, number of abortions, use of condoms, number of sexual partners, number of sexual partners in the past five years and occupation were all associated with female HPV infection rate (P<0.05). The 35–44-year-old women were the age group with the highest HPV infection rate. The HPV infection rate of females in service industries was the highest. Education level and condom use were protective factors of HPV infection, while the number of sexual partners and occupation were risk factors for HPV infection.

## Introduction

Cervical cancer is one of the most common female malignancies. In certain developing countries, cervical cancer has the highest incidence; however, in North America and Europe, the incidence is far lower than the incidences of breast cancer, endometrial cancer and ovarian cancer. According to the estimates of the International Cancer Research Center, there are ∼371,200 new cases of cervical cancer, accounting for ∼9.8% of all tumors and 15% of female cancers annually (1). Of them, 78% of new cases appear in developing countries, while only ∼4.4% occur in developed countries. Annually, there are ∼100,000 new cases of cervical cancer in China, accounting for 1/4 of the global incidence of cervical carcinoma and ranking first in the prevalence of gynecological malignancies (2). In certain developing countries, cervical cancer is the leading cause of mortality for females. In developed countries, the incidence is significantly reduced. In certain countries, the mortality rate of cervical cancer has been reduced by >50% as a result of the early diagnosis and treatment of cervical precancerous lesions (3,4). A number of studies have demonstrated that cervical cancer is associated with human papilloma virus (HPV) infection, particularly the high-risk HPV genotypes (5–7).

At present, a HPV genotyping assay has been adopted in developed regions as a main method for screening cervical lesions. A HPV vaccine is currently the first choice for the prevention of HPV infection and consequent reduction of the incidence of cervical cancers. Research on HPV vaccines is mainly targeted at high-risk HPV genotypes. It is reported that persistent infection with HPV is the crucial factor for cervical cancer (8). To investigate the results of the HPV vaccine and HPV examination-based screening for cervical cancer prevention, HPV infection in large population requires examination.

A preliminary epidemiological study has investigated different genotypes of HPV infection in a number of regions in China (9). However, in Xinjiang, where the incidence of cervical cancer is highest, there have been no region-based population surveys of HPV infection. In the current study, the analysis of female HPV infection in the Urumqi district of Xinjiang was examined, including assessment of age and genotype distribution. This study provides scientific evidence for the HPV vaccine prevention plan and support for an effective cervical cancer prevention strategy.

## Subjects and methods

### Subjects

Females aged 18–69 years in the Urumqi Saybagh district were recruited. All patients provided informed consent. The subjects were divided into four groups, including 543 migrant workers, 525 white-collar workers (government employees, employees in institutionalized organizations and managers in companies), 487 workers in service industries and 714 community residents with a total of 2,269. By the stratified cluster random sampling method, age-stratified sampling was also applied in selected populations, with every 5 years as the age group. Prior written and informed consent were obtained from every patient and the study was approved by the ethics review board of First Affiliated Hospital of Xinjiang Medical University (Urumqi, China).

### Instruments and reagents

A HPV nucleic acid amplification typing detection reagent kit was purchased from Cape Biochemistry Ltd., Co. (Guangdong, China). A HybriMax medical nucleic acid molecule rapid hybridization instrument, an Autocyte Prep system for liquid-based cytology, CytoRich Preservative fluid and an electronic colposcope were purchased from Shenzhen Goldway Industrial Inc. (Shenzhen, China).

### HPV detection

All subjects underwent gynecological examination and cervical secretion was collected. A genome extraction kit from Cape Biochemistry Ltd., Co. was used for DNA extraction. Polymerase chain reaction (PCR) amplification using the ABI7300 PCR amplification instrument (Cape Biochemistry Ltd. Co.) and hybridization were performed. Results were obtained by visual observation. A clear blue purple dot was considered positive. According to the film HPV genotype distribution map, the HPV subtype was determined. If the two control points were positive and other points were negative, DNA test results of HPV subtypes in the reagent kit were considered negative. If at least one HPV genotype point was positive, it demonstrated single or mixed HPV infection.

### Liquid-based cytology test (LCT)

An AutoCyte liquid-based thin-film machine (TriPath Imaging Inc., BD Diagnostics, Burlington, NC, USA) was used for automatic production and dyeing. Results were examined by optical microscopy and cytological diagnosis was in accordance with The Bethesda System (TBS, 2001) (10).

### Colposcopy and pathological diagnosis

For all the subjects with abnormal LCT results, colposcopy and biopsy were performed. For positive high-risk HPV infection in cervical secretions with atypical squamous cells of undetermined sign and/or low-grade squamous intraepithelial lesions in the LCT or worse, colposcopy was performed again. Biopsy was performed by electronic colposcopy in suspicious lesions for pathological diagnosis.

### Statistical analysis

VFP software was used for database building (Microsoft Corp, Redmond, WA, USA). SPSS 16.0 software (SPSS, Inc., Chicago, IL, USA) was used for statistical analysis. Data are presented as the mean ± standard error of the mean (SEM). The HPV infection rates in different age groups with different occupations, cervical intraepithelial neoplasia (CIN; a premalignant cervical disease) rate comparison of different levels and cervical lesion HPV detection rate comparison of different degrees from pathological results were examined by χ^2^ test. Single factor analysis was performed for each factor and analyzed by the logistic regression model. P<0.05 was considered to indicate a statistically significant result.

## Results

### Detected rates of different HPV genotypes in cervical lesions of females of different ages

In this study, 2,269 females were enrolled with an average age of 39.65±0.203 years, ranging from 18 to 69 years. The subjects included 543 migrant workers (individuals going to Urumqi region to work from the other areas of China) with an average age of 35.60±0.612 years, 525 white-collar workers (government employees, employees in institutionalized organizations and managers in companies) with an average age of 34.90±0.319 years, 487 workers in service industries with an average age of 32.78±0.364 years and 714 community residents with an average age of 43.60±0.225 years.

As shown in Table I, there were 460 positive cases of HPV, with a detection rate of 20.27% in the whole investigated group in this study. A total of 21 HPV genotypes were detected, including 13 high-risk genotypes: HPV-16, 18, 31, 33, 35, 39, 45, 51, 52, 56, 58, 59 and 68; five low-risk genotypes: HPV-6, 11, 42, 43, 44; and three common genotypes of Chinese individuals: HPV-53, 56 and CP8304. For the females with infections of high-risk HPV genotypes, the high-risk HPV genotypes were mainly HPV-16, 58, 52 and 18, which accounted for 43.35, 18.35, 8.86 and 7.59%, respectively, of the total infection. The four genotypes accounted for 78.16% of the high-risk HPV genotypes.

**Figure 1.**
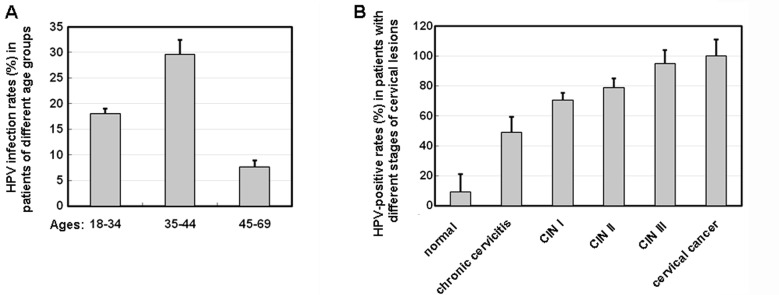
Human papillomavirus (HPV) genotype detection in females aged 18–69 years in Urumqi Saybagh district, China. All subjects underwent gynecological examination and cervical secretion was collected. A genome extraction kit was used for DNA extraction. Polymerase chain reaction (PCR) amplification using the ABI7300 PCR amplification instrument and hybridization were performed. For all the subjects with abnormal liquid-based cytology results, colposcopy and biopsy were performed. The data shown are mean ± standard error of the mean (SEM). (A) HPV infection rates in patients in different age groups. (B) HPV-positive rates in patients with different stages of cervical lesions. Cervical intraepithelial neoplasia (CIN) is a premalignant cervical disease, which includes 3 stages: CIN I, CIN II and CIN III, with increasing disease severity.

As shown in Fig. 1A, the high-risk HPV-positive rates in the three age groups (18–34, 35–44 and 45–69 years) were 18.07, 29.64 and 7.64% (χ^2^=105.02, P<0.05), respectively. The differences in the high-risk HPV-positive rates in the three age groups were significant (χ^2^=19.43, P<0.05; χ^2^=22.62, P<0.01; and χ^2^=66.17, P<0.01, respectively).

### HPV-positive rates are higher among the groups with deteriorating cervical lesions

To determine whether HPV infection is related to different degrees of cervical lesions, HPV infection was determined in the patients with different degrees of cervical lesions. As shown in Fig. 1B, the HPV-positive rate in normal groups without symptoms of cervical lesions was ∼9%. The HPV-positive rates in chronic cervicitis and CIN I patients were ∼49 and 70%, respectively. The HPV-positive rates in CIN II and CIN III patients were ∼79 and 95%, respectively. However, the HPV-positive rate in cervical cancer patients was ∼100%. These results suggest that HPV infection rates are increased in groups with deteriorating cervical lesions (χ^2^=697.72, P<0.05).

### Comparison of the HPV infection rates among different occupation groups

**Figure 2.**
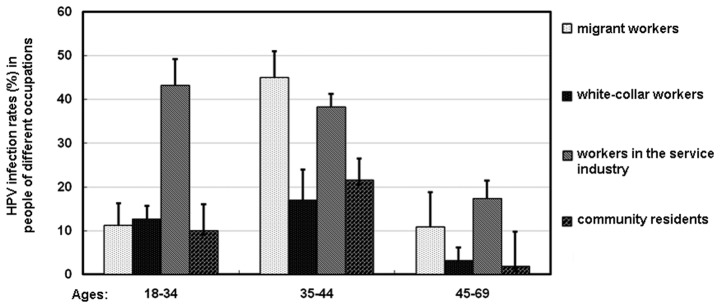
Human papillomavirus (HPV) infection rates of patients (18–69 years old) with different occupations in Urumqi Saybagh district, China. All subjects underwent gynecological examination and cervical secretion was collected. A genome extraction kit was used for DNA extraction. Polymerase chain reaction (PCR) amplification using the ABI7300 PCR amplification instrument and hybridization were performed. For all the subjects with abnormal liquid-based cytology results, colposcopy and biopsy were performed. Data are presented as mean ± standard error of the mean (SEM).

The HPV infection rates in patients with different occupations were also investigated. As shown in Fig. 2, the 35–44-year-old migrant worker group had the highest HPV infection rates among all the groups in the three different age ranges. The HPV infection rate of females in service industries was significantly higher compared with those of the white-collar workers, community residents and migrant worker groups (χ^2^=74.46, P<0.05; χ^2^=84.93, P<0.05; and χ^2^=13.41, P<0.05, respectively) at all three age ranges. These results suggest that the 35–44-year-old migrant worker group and the service industry workers may have a higher risk of HPV infection.

### Analyses of the high-risk HPV infections

To investigate the factors related to the HPV infections, single analysis was performed to study the related factors by comparing data between the HPV-positive and HPV-negative groups. As shown in Table II, several factors, including the number of marriages, education level, smoking history, number of abortions, condom use, number of sexual partners and number of sexual partners within the last 5 years, as well as occupation were significantly different between the two groups (P<0.05; Table II). This analysis indicates that the number of marriages, education level, smoking history, number of abortions, use of condoms, number of sexual partners, number of sexual partners in the past five years and occupation were associated with HPV infection rates (P<0.05).

To further confirm the results, logistic regression model analysis was performed on variables with statistical significance in univariate analysis (α in=0.05, α out=0.10; in, the inclusion criteria; out, the exclusion criteria). The data in Table III shows that education level, number of sexual partners, condom use and occupation were incorporated into the model. The results suggest that education level and condom use were two protective factors of HPV infection, while the number of sexual partners and occupation were risk factors for HPV infection.

## Discussion

In this study, a stratified cross-sectional survey was performed to conduct genotyping detection of 21 types of HPV in a large sample. The distribution of HPV-DNA subtypes varies in different districts and ethnic groups. de Sanjosé *et al* (11) conducted a meta-analysis of HPV infection in six regions of the world in 157,879 females without cervical lesions (by cytological diagnosis). The HPV infection rate was ∼10.4%.

The prevalence of HPV infection varies significantly. The HPV infection rates are as follows: 22.1% in Africa, 20.4% in the central United States and Mexico, 11.3% in North America, 8.1% in Europe and 8.0% in Asia (11). Bell *et al* (12) determined the mean HPV infection rate of 287 American Indian females (by PCR assay) to be 21.25%. Of these, 67.2% was high-risk HPV infection and 41% was multiple infection, and the common HPV infection subtypes were HPV-59, 39 and 73. In China, Li and Dai (13) conducted a study to determine the HPV infection rate in three regions of Shanxi, Shenyang and Shenzhen. The authors identified that the HPV infection rate was 16.1% and HPV-16 is the most common virus type, followed by HPV-58, 52 and 18 (13). In the present study, we identified that the HPV infection rate in Urumqi, Xinjiang is 20.27%, higher than that of Europe and the results of the study by Li and Dai, and similar to that of American Indian females, which may be explained by the fact that Xinjiang is an area with a high incidence of cervical cancer. We demonstrated that the common subtypes in Urumqi, Xinjiang are HPV-16, 58, 52 and 18, which is similar to the results of the study by Li and Dai (8). The existing vaccines are only effective for subtypes 16 and 18; however, 58 and 52 are also highly prevalent. Therefore, a customized HPV vaccine is required for the prevention and treatment of cervical lesions in Chinese females.

In a number of developed countries, including Spain and South Korea, the HPV infection peak usually occurs in young individuals under the age of 25 years and it declines sharply as age increases. In certain areas of South America, the HPV infection rate of 35–54-year-olds is lower than that of younger individuals (<25 years) and elderly individuals (>55 years). In countries with a high incidence of cervical cancer, including India and Nigeria, the HPV infection rate of individuals aged 35–54 years is higher than that in younger individuals (<25 years) and elderly individuals (>55 years) (14). In the present study, we identified that that the infection rate of individuals aged 35–44 years is significantly higher than that in younger individuals (18–34 years) and elderly individuals (>45 years). The infection rate of HPV in the younger individuals was significantly higher than that in the elderly group. Our results are similar to data in India and Nigeria, but different from data in developed countries including Spain and South Korea. The high infection rate in the 35 to 44 age group was possibly due to the reason that females in that age group are sexually active.

HPV infection rates were increased with aggravated cervical lesions. The HPV infection rate in cervical cancer was 100%, demonstrating again the correlation between cervical cancer and HPV infection. HPV infection rates in different age groups with different professions were also examined. The HPV infection rate of workers in service industries was significantly higher than the infection rates of white-collar workers, community residents and migrant workers. Among workers in service industries, the HPV infection rate of females aged 18–34 years was higher than that of those aged 35–44 and 45–69 years. This indicates that female workers in service industries aged 18–34 years are the most sexually active. A lack of health protection is a possible reason for the high HPV infection rate.

In this study, we identified that the number of marriages, education level, smoking history, number of abortions, condom use, number of sexual partners, number of sexual partners in the past five years and occupation are risk factors for HPV infection. A large number of epidemiological studies have shown that a premature sex life, multiple sexual partners, unprotected sexual intercourse and abortion increase the risk of cervical cancer (15–17). One study reported that as the smoking time increases, the risk of cervical cancer increases (18). In the current study, smoking was also observed to be a risk factor for HPV infection. The use of condoms avoids cross-infection, thus reducing the prevalence of HPV.

In conclusion, the infection rate of HPV is high in Urumqi, Xinjiang, with HPV-16, 58, 52 and 18 as the common genotypes. Since females aged 35–44 years have the highest rate of infection, screening of cervical lesions and health habit guidance should be performed.

## Figures and Tables

**Table I.** Distribution of HPV genotypes infecting females in Urumqi.

HPV genotype	Total positive cases	Positive ratio of population^a^	Positive ratio of group^a^
High-risk			
HPV-16	137	137/2269 (6.03)	137/316 (43.35)
HPV-18	24	24/2269 (1.05)	24/316 (7.59)
HPV-31	21	21/2269 (0.93)	21/316 (6.65)
HPV-33	13	13/2269 (0.57)	13/316 (4.11)
HPV-35	0	0	0
HPV-39	10	10/2269 (0.44)	10/316 (3.16)
HPV-45	5	5/2269 (0.22)	5/316 (1.58)
HPV-51	5	5/2269 (0.22)	5/316 (1.58)
HPV-52	28	28/2269 (1.23)	28/316 (8.86)
HPV-56	5	5/2269 (0.22)	5/316 (1.58)
HPV-58	58	58/2269 (2.56)	58/316 (18.35)
HPV-59	0	0	0
HPV-68	10	10/2269 (0.44)	10/316 (3.16)
Low-risk			
HPV-6	5	5/2269 (0.22)	5/14 (35.71)
HPV-11	3	3/2269 (0.13)	3/14 (21.43)
HPV-42	3	3/2269 (0.13)	3/14 (21.43)
HPV-43	3	3/2269 (0.13)	3/14 (21.43)
HPV-44	0	0	0
Common type for Chinese			
HPV-53	10	10/2269 (0.44)	10/30 (33.33)
HPV-66	10	10/2269 (0.44)	10/30 (33.33)
HPV-CP8304	10	10/2269 (0.44)	10/30 (33.33)
Mixed infection	100	100/2269 (4.41)	100/100 (100.0)
Total	460		

a Values in brackets are percentages. HPV, human papillomavirus.

**Table II.** Single factor analysis of the risk factors of HPV infection.

Variables	HPV-positive cases	HPV-negative cases	OR value	95% CI
Number of marriages				
0	70	133	1	
1	471	1405	0.632^a^	0.408–1.001
≥2	93	97	1.786^a^	1.022–3.369
Education level				
Junior high school or below	464	863	1	
High school or above	169	773	0.369^a^	0.304–0.601
Married				
Yes	551	1445	1	
No	82	191	0.854	0.534–1.313
History of cervical disease				
Yes	384	982	1	
No	250	653	1.019	0.765–1.343
Tuberculosis				
Yes	4	8	1	
No	630	1627	1.289	0.231–7.083
History of STDs				
Yes	25	55	1	
No	609	1580	1.187	0.587–2.402
History of smoking				
No	303	904	1	
Yes	331	731	1.349^a^	1.039–1.771
Age of first menstruation				
≤11 years	106	297	1	
12–15 years	432	1078	1.133	0.791–1.622
≥16 years	95	261	1.041	0.641–1.649
Menopause				
Yes	93	233	1	
No	541	1402	1.032	0.712–1.523
Abortion				
Yes	74	231	1	
No	560	1404	0.811	0.532–1.201
Number of abortions				
1	184	598	1	
2	237	545	1.415^a^	1.024–1.961
≥3	212	493	1.406^a^	1.001–1.972
Condom use				
Yes	216	666	1	
No	418	969	1.334^a^	1.005–1.761
Number of sexual partners				
1	500	1430	1	
2	91	140	1.855^a^	1.231–2.807
≥3	42	66	1.901^a^	1.603–3.422
Number of sexual partners in the past 5 years				
1	479	1366	1	
2	87	157	1.577^a^	1.042–2.376
≥3	67	113	1.749^a^	1.103–2.789
Sexual intercourse outside marriage				
Yes	50	140	1	
No	583	1496	0.933	0.569–1.523
Age at first sexual intercourse				
≤18 years	129	295	1	
19–22 years	224	668	0.761	0.524–1.121
≥23 years	280	673	0.931	0.637–1.336
Occupation				
Community residents	91	623	1	
White-collar workers	64	461	1.902	0.831–1.743
Migrant workers	134	409	3.288^a^	2.241–4.851
Workers in service industry	171	316	3.995^a^	2.473–5.247

a P<0.05. HPV, human papillomavirus; OR, odds ratio; CI, confidence interval; STDs, sexually transmitted diseases.

**Table III.** Multiple factor analysis of the risk factors of HPV infection.

Factor	B	SE	Wald	df	P-value	Exp(B)	95% CI for Exp(B)	
							Lower	Upper
Education level	−0.871	0.152	32.233	1	0.000	0.423	0.301	0.559
Number of sexual partners	0.326	0.133	5.923	1	0.012	1.379	1.062	1.791
Condom use	−0.372	0.146	6.238	1	0.009	0.681	0.509	0.919
Occupation	0.453	0.061	51.949	1	0.000	1.581	1.389	1.778

B, partial regression coefficient; Wald, Wald χ^2^ test; df, degree of freedom; Exp (B), exponentiation of the B coefficient; CI, confidence interval; HPV, human papillomavirus.
